# Upregulation of nAChRs and Changes in Excitability on VTA Dopamine and GABA Neurons Correlates to Changes in Nicotine-Reward-Related Behavior

**DOI:** 10.1523/ENEURO.0189-20.2020

**Published:** 2020-10-14

**Authors:** Austin T. Akers, Skylar Y. Cooper, Zachary J. Baumgard, Gabriella P. Casinelli, Alicia J. Avelar, Brandon J. Henderson

**Affiliations:** Department of Biomedical Sciences, Marshall University, Joan C Edwards School of Medicine, Huntington, WV 25703

**Keywords:** excitability, nicotine, nicotinic receptor, reward, upregulation

## Abstract

Previous reports indicate that nicotine reward is mediated through α4β2*, α6β2*, and α4α6β2* nicotinic acetylcholine receptors (nAChRs; * indicates that additional nAChR subunits may be present). Little is known about α4α6β2* nAChR involvement in reward and reinforcement because of a lack of methods that allow the direct investigation of this particular nAChR subtype. Here, we use male and female mice that contain α4-mCherry and α6-GFP nAChR subunits to show that concentrations of nicotine sufficient to evoke reward-related behavior robustly upregulate α4* and α4α6* nAChRs on midbrain dopamine (DA) and GABA neurons. Furthermore, the extent of α4α6* nAChR upregulation on ventral tegmental area (VTA) DA neurons aligns with the magnitude of nicotine reward-related behavior. We also show that the upregulation of nAChRs is accompanied by a functional change in firing frequency of both DA and GABA neurons in the VTA that is directly linked to nicotine reward-related behavior.

## Significance Statement

This study reveals novel aspects of nicotinic acetylcholine receptor upregulation in the ventral tegmental area (VTA) and how it is linked to nicotine reward-related behavior. Nicotine is well characterized to upregulate these receptors, but our work here highlights the role that α4* and α4α6* nicotinic acetylcholine receptors (nAChRs) play in the short term, intermittent exposure to nicotine that is consistent with early exposure to tobacco and vaping products.

## Introduction

The use of tobacco or nicotine-containing products remains the leading cause of preventable death. Each year, nearly 6 million people die worldwide because of tobacco-related health complications (World Health Organization, 2011). In the United States alone, ∼480,000 people die annually from tobacco products resulting in $75 billion in medical costs [National Center for Chronic Disease Prevention and Health Promotion (US) Office on Smoking and Health, 2014]. Nicotine, the primary addictive component of tobacco products, stimulates dopamine (DA) neurotransmission through the activation of nicotinic acetylcholine receptors (nAChRs). Several nAChR subtypes contribute to the multiple stages of nicotine addiction, but many reports highlight α4β2*, α6β2β3, and α4α6β2* nAChRs as primary mediators of nicotine reward, because these subtypes exhibit the highest sensitivity to acutely applied nicotine (Kuryatov and Lindstrom, 2011; Liu et al., 2012; Engle et al., 2013). Furthermore, studies have shown that these nAChRs on VTA DA neurons are essential for intravenous self-administration of nicotine (Pons et al., 2008).

One well-studied phenomenon of α4* (and in some cases α6*) nAChRs is that they upregulate following long-term exposure to nicotine (Marks et al., 1983; Nashmi et al., 2007; Jasinska et al., 2014; Henderson et al., 2016). These upregulated nAChRs exhibit an increase in sensitivity to nicotine (Kuryatov et al., 2005; Vallejo et al., 2005). In VTA DA neurons, α4β2*, α6β2*, and α4α6β2* nAChRs are upregulated by nicotine and the amount of upregulation depends on the concentration of nicotine used (Nashmi et al., 2007; Henderson et al., 2017). In ventral tegmental area (VTA) and substantia nigra pars reticulata (SNr) GABA neurons, α4β2 nAChRs are upregulated more robustly than nAChRs present on VTA DA neurons (Nashmi et al., 2007). The upregulated α4β2 nAChRs on GABA neurons exhibit increased sensitivity to nicotine reward (Nashmi et al., 2007; Ngolab et al., 2015) and are rapidly desensitized during acute nicotine exposure (Mansvelder et al., 2002), resulting in the disinhibition of VTA DA neurons and an increase in DA neurotransmission within the mesolimbic pathway. While the inhibitory inputs to VTA DA neurons are altered by nicotine, there is also an enhancement in excitatory inputs from medial VTA glutamate neurons (Yan et al., 2018, 2019). Together, these changes in inhibitory and excitatory transmission contribute to nicotine reward.

Despite general agreement that long-term nicotine exposure produces nAChR upregulation, there is little understanding regarding how nAChR upregulation is related to behaviors associated with nicotine reward or reinforcement. We sought to fill this gap in knowledge by assessing the magnitude of nAChR upregulation in mice that completed conditioned place preference (CPP) assays with nicotine. To accomplish this we used a previously characterized mouse line that contain α4-mCherry and α6-GFP nAChR subunits (Henderson et al., 2017). In using the same mice for both CPP and nAChR upregulation assays, we were able to establish direct links between nicotine reward-related behavior and nAChR upregulation.

## Materials and Methods

### Reagents

(–)-Nicotine dihydrogen ditartrate (product number AC415660100) was obtained from ACROS Organics. All doses and concentrations of nicotine listed here are given as free base.

### Mice

All experiments were conducted in accordance with the guidelines for care and use of animals provided by the National Institutes of Health, and protocols were approved by the Institutional Animal Care and Use Committee at Marshall University. Mice were kept on a standard 12/12 h light/dark cycle at 22°C and given food and water *ad libitum*. At day 21, tail biopsies were taken for genotyping analysis by PCR. Genotyping was outsourced to a commercial lab to detect the presence of fluorescence proteins (Transnetyx). For microscopy assays, only mice that were transgenic for α6-GFP and homozygous for α4-mCherry were used (see CPP assay methods below), with the exception of α6-GFP and α4-mCherry mice used for NFRET controls. For electrophysiology assays we used α6-GFP mice. All experiments used adult (three to five months old) mice. Both male and female mice were used and numbers of each are detailed below in the methods for specific experiments.

### CPP assays

CPP assays were completed with a three-chamber spatial place-preference chamber (Harvard Apparatus, PanLab) (Fig. 2). Time in chambers was recorded by video motion tracking software (SMART 3.0). An unbiased protocol was used where nicotine was given in the white/gray chamber on “drug” days, and saline was given in the white/black chamber on saline days. For saline-control cohorts, saline injections were given on both sides. On day 1 of testing, mice were placed in the central chamber and allowed free access for 20 min. Mice that spent >65% of the test in one chamber were judged to show initial biases and were excluded from the experiment. On days 2–5, mice were trained using twice-daily sessions. In morning sessions, mice were given an intraperitoneal injection of drug (saline or 0.5 mg/kg nicotine) immediately before confinement in the drug-paired chamber for 20 min. In the afternoon session, mice were given an intraperitoneal injection of saline immediately before confinement in the saline-paired chamber for 20 min. On day 6, a post-test was completed where the mice were again placed in the central chamber and allowed free access for 20 min. A total of 25 mice were used in CPP assays, and two were excluded because of initial bias. Nearly equal numbers of male and female mice, three to six months old, were used in CPP assays. No sex differences were observed at this dose of nicotine. Data are expressed as a change in baseline preference between the pre-test and post-test. Of these mice, 12 were later studied in confocal microscopy assays (see below).

Between the A.M. and P.M. CPP sessions, each mouse experienced a break of ∼4 h. Given the pharmacokinetics of nicotine, injections up to 1 mg/kg are cleared in ≤1 h and most likely within 30 min (Matta et al., 2007; Li et al., 2013). Thus, nicotine is not present in the brain or plasma during the PM CPP saline conditioning sessions. For each cohort, only mice of the same sex were tested. All drugs used in CPP assays were dissolved in saline at pH 7.4 and injected intraperitoneally.

### Confocal imaging of mouse brain slices

Some mice studied in CPP assays were also studied in microscopy assays. Drug delivery for these cohorts is described in the above CPP section. Following the completion of CPP assays, mice were euthanized with CO_2_ and subjected to a quick cardiac perfusion with 10-ml ice-cold saline. We found that this significantly reduced the autofluorescence in the mCherry emission range. The brain was then quickly removed and frozen using acetone and dry ice and then stored at −80°C overnight. At least 24 h later, brains were sectioned at 20 μm using a cryostat onto glass slides (1.0 mm). Coronal brain slices were mounted with Vectashield (Vector Labs, H-1000) and coverslipped (1.5-mm thickness, glass). We targeted bregma −3.1 mm because this region gave the most consistent sections that contained a large portion of the VTA, SNr, substantia nigra pars compacta (SNc), and dentate gyrus in a single slice. In each session, brain slices from mice that received different drug treatments were selected according to bregma (−3.1 mm) so that all imaging sessions were matched between animals and across cohorts.

A Leica SP5 TCSII confocal microscope was used for imaging of the α4-mCherryα6-GFP mouse brain slices. α6-GFP and α4-mCherry were excited at 488 and 561 nm, respectively; 40× images with a 5× digital zoom were collected for the quantitative measurements of α4-mCherry and α6-GFP neuron raw integrated density (RID) of the midbrain, while 10× images with a 5× digital zoom were collected for the quantitative measurements of α4-mCherry expression of the dentate gyrus. NFRET was calculated using the PixFRET ImageJ plug-in. Control data for NFRET donor and acceptor bleedthrough were obtained by imaging neurons from homozygous α4-mCherry mice (wild-type α6) and α6-GFP (wild-type α4) mice separately. In total, 19 male and 13 female mice were used in confocal assays following CPP (excluding those used for NFRET controls). Nearly equal numbers of male and female mice, three to five months old, were used and no sex differences were observed.

All calculations and analysis of GFP and mCherry fluorescence were made using ImageJ. We used RID as a metric for upregulation to match similar work on nAChR upregulation (Avelar et al., 2019; Cooper et al., 2020). For each mouse, two to three different brain slices were imaged by two to three experimenters blind to drug treatment until all data analysis was completed. For each brain slice, 41–71 VTA DA neurons, 36–50 VTA GABA neurons, or 36–68 SNr GABA neurons were imaged. Data were averaged to provide RID values for each mouse. To minimize biasing, we did not select subpopulations in each brain region and analyzed every GFP and/or mCherry neuron present in each brain area.

### Calculation of upregulation score

Following microscopy assays of saline-treated and nicotine-treated mice from CPP assays, we calculated the mean RID for α4* (α4-mCherry), α6* (α6-GFP), and α4α6* (identified by NFRET) nAChRs in saline-treated mice (*n* = 6 mice). We subtracted these mean RID values from the RID of individual mice used in the nicotine CPP cohort to obtain the upregulation score. For example, the upregulation scores for a hypothetical mouse X are calculated as following:

$$\alpha 4mCherry_{UpregScore}^{MouseX}=\alpha 4mCherry_{NicotineTreatedRID}^{MouseX}-MEAN\alpha 4mCherry_{SalineTreatedRID}^{Mice1-6}$$

$$\alpha 6GFP_{UpregScore}^{MouseX}=\alpha 6GFP_{NicotineTreatedRID}^{MouseX}-MEAN\alpha 6GFP_{SalineTreatedRID}^{Mice1-6}$$

$$\alpha 4\alpha 6_{UpregScore}^{MouseX}=\alpha 4\alpha 6_{NicotineTreatedRID}^{MouseX}-MEAN\alpha 4\alpha 6_{SalineTreatedRID}^{Mice1-6}.$$

These upregulation scores are plotted as *y*-axis values with the mouse’s respective CPP scores as the *x*-axis values (see Figs. 3, 4). For linear regression analysis, we used GraphPad Prism software with no constraints on the data. This analysis in GraphPad provided *R*^2^, *F*, and *p* values.

### Immunofluorescence

**Figure 1.**
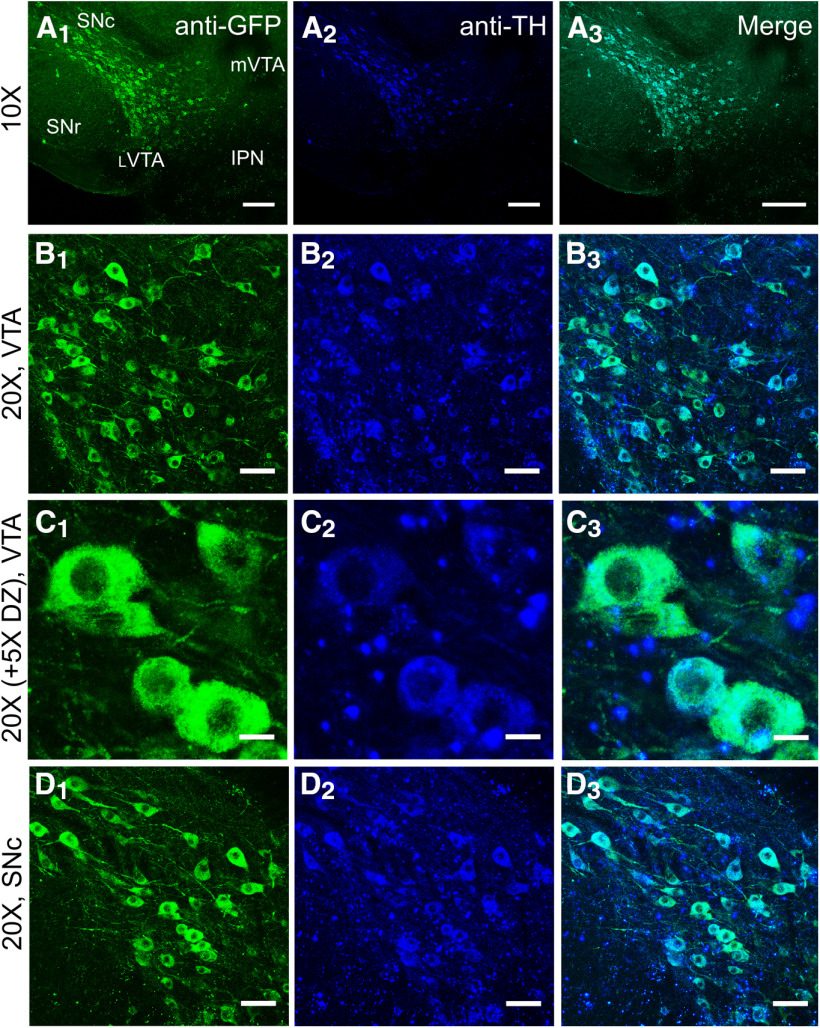
Presence of α6-GFP overlaps with presence of TH. ***A_1_–A_3_***, 10× images of an α6-GFP mouse coronal brain slice (bregma, −3.1) immunostained with anti-GFP/Alexa Fluor 488 and anti-TH/Alexa Fluor 647. ***B***, 20× image of neurons in the _L_VTA displaying overlap of α6-GFP and TH presence. ***C***, 20× (with 5× digital zoom) images of _L_VTA. ***D***, 20× Images of SNc neurons. Scale bars: 100 μm (***A_1_–A_3_***), 50 μm (***B_1_–B_3_***, ***D_1_–D_3_***), and 10 μm (***C_1_–C_3_***). *Figure Contributions*: Brandon J. Henderson performed the experiments.

For immunofluorescence assays (Fig. 1 only), we used three-month-old α6-GFP mice. Mice were anesthetized in a manner identical to the method used for microscopy. However, before brain extraction, mice were perfused with 20-ml PBS and 40 ml 4% paraformaldehyde. Brains were extracted and placed in 40% sucrose for 24 h. Brains were sliced at a thickness of 20 μm and mounted on microscope slides. Brain sections on slides were rinsed twice for 10 min with PBS and then permeabilized with 0.5% Triton X-100 in PBS for 1 h. Brain sections were then blocked with 4% goat serum in PBS for 45 min. The primary antibody was diluted in 4% goat serum in PBS and incubated overnight at 24°C [rabbit anti-GFP (A11122, Invitrogen) and sheep anti-TH (AB1542, Millipore) both at concentrations of 1:500]. Brain sections were then washed three times for 15 min with PBS, and the secondary antibody was diluted in 4% goat serum in PBS and incubated for 1 h at 24°C [goat anti-rabbit Alexa Fluor 488 (A11008, Invitrogen) and donkey anti-sheep Alexa Fluor 647 (ab150179, Abcam) both at concentrations of 1:1000]. Brain sections were washed three times for 15 min with PBS and mounted with Vectashield. Finally, brain slices were imaged using the same confocal microscope used for direct imaging of α4-mCherryα6-GFP mouse brain slices as described above.

### Whole-cell patch-clamp electrophysiology

Recordings were performed using brain slices prepared from three-month-old male α6-GFP mice. We used α6-GFP mice because α6 nAChR subunits are selectively expressed in DA neurons in the lateral VTA (_L_VTA) and SNc and provide a method to identify putative DA (pDA) neurons (see Fig. 1). Previous investigations have also concluded that α6-GFP expression in the _L_VTA and SNc completely overlaps with the expression of tyrosine hydroxylase (TH; Mackey et al., 2012). To match the investigation of VTA DA neurons in microscopy assays, we targeted bregma −3.1 for electrophysiology assays. Mice were anesthetized with CO_2_ and were subjected to cardiac perfusion. The artificial cerebral spinal fluid solutions we used were made in accordance to the protective recovery method (www.brainslicemethods.com). We used standard artificial CSF (ACSF; containing the following: 125 mm NaCl, 2.5 mm KCl, 1.2 mm NaH_2_PO_4_, 1.2 mm MgCl_2_, 2.4 mm CaCl_2_, 26 mm NaHCO_3_, and 11 mm glucose, adjusted to 300–310 mOsm and pH 7.4) for transcardial perfusion and brain slicing (250-μm coronal sections). Following cutting, brain slices containing VTA DA neurons were allowed to recover at 32–34°C in a NMDG ACSF solution (containing the following: 93 mm NMDG, 2.5 mm KCl, 1.2 mm NaH_2_PO_4_, 30 mm NaHCO_3_, 20 mm HEPES, 25 mm glucose, 5 mm Na-ascorbate, 2 mm thiourea, 3 mm Na-pyruvate, 10 mm MgSO_4_, and 0.5 mm CaCl_2_, adjusted to 300–310 mOsm and pH 7.4) for 15 min. Next, brain slices remained in standard ACSF at room temperature for ≥60 min. Slices were then transferred to the slice chamber and continually perfused with carbogen-saturated standard ACSF (1.5–2.0 ml/min) at 32°C.

Neurons were visualized with an upright microscope (Axio Examiner A1, Zeiss) equipped with an Axiocam 702 mono using DIC near-infrared illumination. Blue illumination was used to visualize α6-GFP presence in pDA neurons. Cell-attached and whole-cell patch-clamp techniques were used to record electrophysiological signals with an Integrated Patch-Clamp Amplifier (Sutter). Patch electrodes had resistances of 4–10 MΩ when filled with intrapipette solution: 135 mm K gluconate, 5 mm KCl, 5 mm EGTA, 0.5 mm CaCl_2_, 10 mm HEPES, 2 mm Mg-ATP, and 0.1 mm GTP (adjusted to 280–300 mOsm and pH of 7.4). Recordings were sampled at 10–40 kHz. The junction potential between patch pipette and bath solutions was nulled just before gigaseal formation. Series resistance was monitored without compensation throughout experiments using SutterPatch software. The recording sessions for neurons were terminated if the series resistance changed by >20%. Nicotine (hydrogen tartrate salt, dissolved in standard ACSF at pH 7.4) applications were applied using a Picospritzer III (Parker) at a pressure of 5 psi for 20 s at concentration of 300 and 500 μm.

### Statistical analysis

All results are presented as mean ± SEM, and all statistical analyses were performed using GraphPad Prism. For CPP assays, upregulation, and comparison of firing frequencies, we used an unpaired, two-tailed *t* test to compare saline-treated and nicotine-treated groups. For correlations between CPP scores and nAChR upregulation, we used GraphPad Prism software to perform linear regression analysis. All statistical test results are presented in Table 1.

**Table 1:** Stats table

Figure	Figure panel	Statistical test	Conditions	Results
2	*C*	Two-way ANOVA interaction		*F*_(1,35)_ = 0.016 *p *=* *0.90
		Two-way ANOVA sex factor		*F*_(1,35)_ = 0.02 *p *=* *0.89
		Two-way ANOVA drug factor		*F*_(1,35)_ = 23.9 *p *=* *0.00002
		Two-way ANOVA means comparison	Male mice (CPP, saline vs nicotine)	*p *=* *0.0011
		Two-way ANOVA means comparison	Female mice (CPP, saline vs nicotine)	*p *=* *0.0064
	*D_1_*	Two-way ANOVA interaction	α4α6* nAChR upregulation	*F*_(1,23)_ = 0.45 *p *=* *0.51
		Two-way ANOVA sex factor	α4α6* nAChR upregulation	*F*_(1,25)_ = 0.12 *p *=* *0.73
		Two-way ANOVA drug factor	α4α6* nAChR upregulation	*F*_(1,25)_ = 19.0 *p *=* *0.0002
		Two-way ANOVA means comparison	Male mice (α4α6* RID, saline vs nicotine)	*p *=* *0.019
		Two-way ANOVA means comparison	Female mice (α4α6* RID, saline vs nicotine)	*p *=* *0.0054
	*D_2_*	Two-way ANOVA interaction	α4* nAChR upregulation	*F*_(1,25)_ = 0.74 *p *=* *0.40
		Two-way ANOVA sex factor	α4* nAChR upregulation	*F*_(1,25)_ = 0.30 *p *=* *0.59
		Two-way ANOVA drug factor	α4* nAChR upregulation	*F*_(1,25)_ = 15.5 *p *=* *0.0005
		Two-way ANOVA means comparison	Male mice (α4* RID, saline vs nicotine)	*p *=* *0.05
		Two-way ANOVA means comparison	Female mice (α4* RID, saline vs nicotine)	*p *=* *0.008
	*D_3_*	Two-way ANOVA interaction	α6* nAChR upregulation	*F*_(1,25)_ = 0.32 *p *=* *0.58
		Two-way ANOVA sex factor	α6* nAChR upregulation	*F*_(1,25)_ = 0.87 *p *=* *0.36
		Two-way ANOVA drug factor	α6* nAChR upregulation	*F*_(1,25)_ = 0.086 *p *=* *0.77
		Two-way ANOVA means comparison	Male mice (α6* RID, saline vs nicotine)	*p *=* *0.99
		Two-way ANOVA means comparison	Female mice (α6* RID, saline vs nicotine)	*p *=* *0.99
3	*A_2_*	Linear regression	Male mice, CPP score vs VTA pDA α4α6* nAChR upregulation	*R*^2^ = 0.67 *F*_(1,5)_ = 10.1 *p *=* *0.025
	*A_3_*	Linear regression	Male mice, CPP score vs VTA pDA α4* nAChR upregulation	*R*^2^ = 0.83 *F*_(1,5)_ = 24.4 *p *=* *0.004
	*A_4_*	Linear regression	Male mice, CPP score vs VTA pDA α6* nAChR upregulation	*R*^2^ = 0.74 *F*_(1,5)_ = 14.3 *p *=* *0.013
	*B_1_*	Linear regression	Female mice, CPP score vs VTA pDA α4α6* nAChR upregulation	*R*^2^ = 0.82 *F*_(1,6)_ = 28.2 *p *=* *0.002
	*B_2_*	Linear regression	Female mice, CPP score vs VTA pDA α4* nAChR upregulation	*R*^2^ = 0.81 *F*_(1,6)_ = 25.6 *p *=* *0.002
	*B_3_*	Linear regression	Female mice, CPP score vs VTA pDA α6* nAChR upregulation	*R*^2^ = 0.07 *F*_(1,6)_ = 0.47 *p *=* *0.52
	*C_2_*	Linear regression	CPP score vs VTA pDA α4α6* nAChR upregulation	*R*^2^ = 0.08 *F*_(1,3)_ = 0.24 *p *=* *0.65
	*C_3_*	Linear regression	CPP score vs VTA pDA α4* nAChR upregulation	*R*^2^ = 0.08 *F*_(1,3)_ = 0.26 *p *=* *0.64
	*C_4_*	Linear regression	CPP score vs VTA pDA α6* nAChR upregulation	*R*^2^ < 0.01 *F*_(1,3)_ = 0.01 *p *=* *0.94
4	*A_2_*	Two-way ANOVA interaction	α4* nAChR upregulation	*F*_(1,27)_ = 0.99 *p *=* *0.33
		Two-way ANOVA sex factor	α4* nAChR upregulation	*F*_(1,27)_ = 2.86 *p *=* *0.10
		Two-way ANOVA drug factor	α4* nAChR upregulation	*F*_(1,27)_ = 17.1 *p *=* *0.0003
		Two-way ANOVA means comparison	Male mice (α4* RID, saline vs nicotine)	*p *=* *0.0012
		Two-way ANOVA means comparison	Female mice (α4* RID, saline vs nicotine)	*p *=* *0.05
	*A_3_*	Linear regression	Male CPP score vs SNr GABA α4* nAChR upregulation	*R*^2^ = 0.55 *F*_(1,6)_ = 7.39 *p *=* *0.04
	*A_4_*	Linear regression	Female CPP score vs SNr GABA α4* nAChR upregulation	*R*^2^ = 0.56 *F*_(1,6)_ = 6.45 *p *=* *0.05
	*B_2_*	Two-way ANOVA interaction	α4* nAChR upregulation	*F*_(1,24)_ = 0.43 *p *=* *0.52
		Two-way ANOVA sex factor	α4* nAChR upregulation	*F*_(1,24)_ = 0.38 *p *=* *0.55
		Two-way ANOVA drug factor	α4* nAChR upregulation	*F*_(1,24)_ = 14.0 *p *=* *0.001
		Two-way ANOVA means comparison	Male mice (α4* RID, saline vs nicotine)	*p *=* *0.04
		Two-way ANOVA means comparison	Female mice (α4* RID, saline vs nicotine)	*p *=* *0.013
	*B_3_*	Linear regression	Male CPP score vs SNr GABA α4* nAChR upregulation	*R*^2^ < 0.001 *F*_(1,6)_ < 0.001 *p *=* *0.99
	*B_4_*	Linear regression	Female CPP score vs SNr GABA α4* nAChR upregulation	*R*^2^ = 0.075 *F*_(1,6)_ = 0.40 *p *=* *0.55
	*D_1_*	Unpaired *t* test	Dentate Gyrus α4* nAChR upregulation, saline vs nicotine	*p *=* *0.047
	*D_2_*	Linear regression	CPP score vs dentate gyrus α4* nAChR upregulation	*R*^2^ = 0.02 *F*_(1,4)_ = 0.09 *p *=* *0.78
5	*C*	Unpaired *t* test	CPP score, saline vs nicotine	*p *=* *0.026
	*E*	Unpaired *t* test	VTA pDA neuron firing frequency, saline vs nicotine	*p *=* *0.0094
	*F*	Linear regression	CPP Score vs VTA pDA neuron firing frequency	*R*^2^ = 0.65 *F*_(1,16)_ = 11.2 *p *=* *0.016
6	*D*	Unpaired *t* test	VTA pGABA neuron firing frequency, saline vs nicotine	*p *=* *0.0056
	*E*	Linear regression	CPP Score vs VTA pGABA neuron firing frequency	*R*^2^ = 0.77 *F*_(1,16)_ = 20.3 *p *=* *0.004

## Results

### Nicotine reward-related behavior in α4-mCherryα6-GFP mice

Given that our goal was to investigate a link between nAChR upregulation and nicotine reward-related behavior, we required a method to reliably identify DA and GABA neurons in the VTA. To distinguish between DA and GABA neurons in the VTA, we used the presence of α6-GFP nAChRs in a manner that is similar to previous reports that have used fluorescently tagged nAChR subunits (Henderson et al., 2014, 2016, 2017; Cooper et al., 2020). Previous investigations have shown that α6-GFP or β3-GFP nAChRs that are selectively expressed in VTA and SNc DA neurons exhibit a high co-localization with TH presence (>95%; Mackey et al., 2012; Srinivasan et al., 2016). To confirm this using our own methods, we used immunofluorescence on coronal brain slices (bregma, −3.1) from α4-mCherryα6-GFP mice to examine the co-localization of α6-GFP in this mouse line with TH (Fig. 1). Here, we observed that 100% of the α6-GFP-labeled neurons in the _L_VTA and SNc co-localized with TH (Fig. 1; Table 2). Conversely, 83.9% and 73.9% of the TH-positive neurons contained α6-GFP in the _L_VTA and SNc, respectively (Fig. 1; Table 2). We acknowledge that α6 nAChR subunits are expressed on GABAergic boutons in the VTA (Yang et al., 2011). Accordingly, we have assigned neurons positive for α6-GFP expression as pDA neurons.

**Table 2:** Immunofluorescence

	_L_VTA	SNc
Number of TH+	768	184
Number of GFP+	644	136
% of GFP+ to TH+	83.9%	73.9%

All GFP+ neurons overlapped with TH+ neurons.

**Figure 2.**
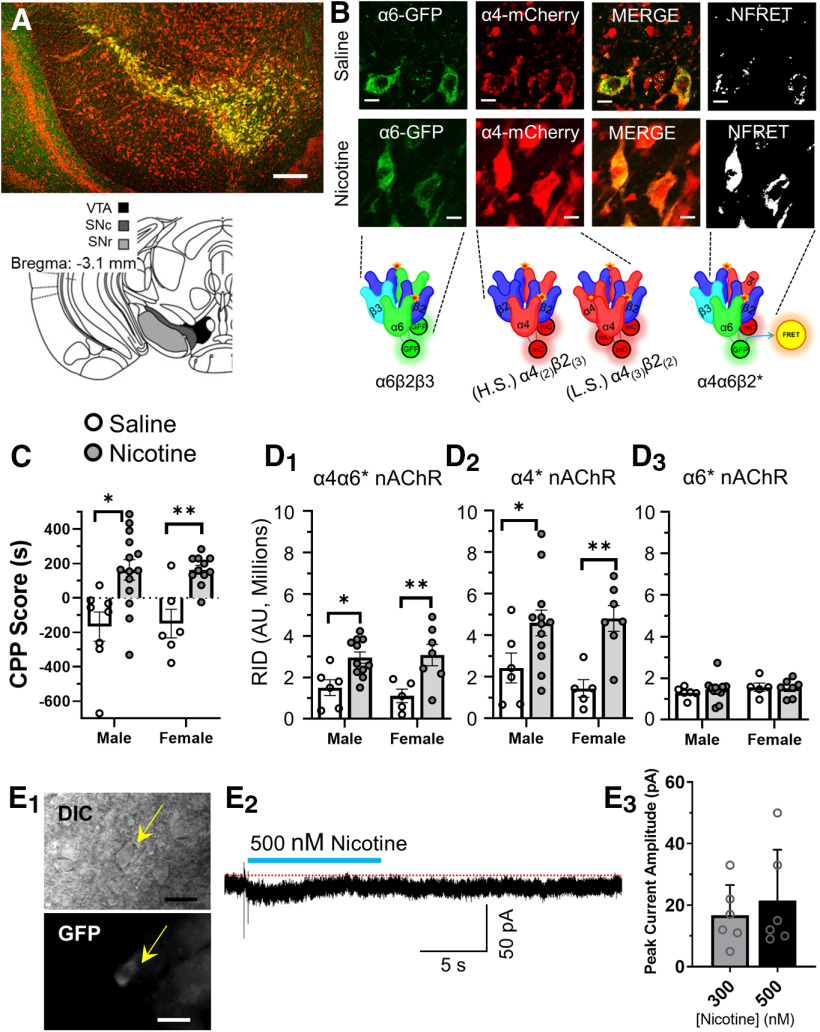
α4-mCherryα6-GFP mice reveal multiple subtypes of nAChRs on VTA pDA neurons. ***A***, Schematic of target mouse brain region (bottom) and sample 10× image of a mouse coronal brain slice at approximately bregma −3.1 mm (top, no immunofluorescence was used here). Scale bar: 250 μm. ***B***, Sample images of control and nicotine-treated VTA DA neurons, set to the same intensity scale, from α4-mCherryα6-GFP mice used in a CPP assay. Scale bar: 10 μm. ***C***, Male and female mice were administered intraperitoneal injections of saline or 0.5 mg/kg nicotine in a CPP assay [*n* = 14 (8 males and 6 females) and 25 (14 males and 11 females) for saline and nicotine, respectively]. ***D_1_–D_3_***, RID of α4α6*, α4*, and α6* nAChRs in saline-treated and nicotine-treated mice (from CPP assays). Individual dots represent the RID of individual mice [*n* = 11 (6 males and 5 females) and 19 (12 males and 7 females) for saline and nicotine, respectively]. For each mouse, 41–71 putative _L_VTA DA neurons were imaged. ***E_1_***, Representative image of a putative VTA DA neuron in a brain slice (bregma, −3.1) from an α6-GFP mouse (scale bars: 20 μm). ***E_2_***, Representative voltage-clamp recording of putative _L_VTA DA neurons during a 10-s puff of 300 and 500 nm nicotine. Blue bar indicates duration of nicotine puff and red dotted line represents baseline before nicotine puff. All data are mean ± SEM; *p < 0.05, **p < 0.05, ***p < 0.005; unpaired, two-tailed *t* test. Exact *p* values are given in Results. *Figure Contributions*: Austin T. Akers, Zachary J. Baumgard, Skylar Y. Cooper, Gabriella P. Casinelli, Alicia J. Avelar, and Brandon J. Henderson performed the experiments and analyzed the data.

The α4-mCherryα6-GFP mice enable the examination of α4β2*, α6β2*, and α4α6β2* nAChRs in midbrain DA and GABA neurons (Fig. 2*A*,*B*; Henderson et al., 2017). We tested mice using a CPP assay to measure reward-related behavior (Fig. 2*C*). In rodents, acute nicotine produces rewarding effects depending on route of administration and concentration. Previous investigations have reported that mice exhibit reward-related behavior to 0.5 mg/kg nicotine in a CPP assay (Tapper et al., 2004; Sanjakdar et al., 2015; Henderson et al., 2016, 2017). In agreement with these previous reports, male and female α4-mCherryα6-GFP mice exhibited significant reward-related behavior to intraperitoneal injections of 0.5 mg/kg nicotine when compared with saline (*p = *0.0011 and *p = *0.006, respectively; Fig. 2*C*). No sex differences were noted with this single dose of nicotine (Table 1), but we acknowledge this may not be the case if the dose range were to be expanded.

Our CPP experiments used intraperitoneal injections of 0.5 mg/kg nicotine. Based on nicotine pharmacokinetics in mice (Matta et al., 2007), these daily injections produced a “peak” brain concentration of ∼250 nm nicotine that would be eliminated in ∼30 min. This would be applicable to beginning smokers in the early stages of dependence (Tapper et al., 2004). It is important to note that nAChRs on VTA DA neurons would be functionally activated at this concentration of nicotine as previous investigations show that 300 nm nicotine is sufficient to evoke inward currents in VTA DA neurons (Liu et al., 2012; Engle et al., 2013). These previous reports also stress that this low concentration of nicotine only activates nAChRs that contain both α4 and α6 nAChR subunits. To confirm this, we conducted our own whole-cell patch-clamp electrophysiology assays to determine whether 300 and 500 nm nicotine stimulates inward currents in VTA DA neurons. We prepared brain slices from three-month-old male α6-GFP mice (target bregma, −3.1). VTA pDA neurons were identified by presence of α6-GFP (Fig. 2*E1*), firing frequencies <10 Hz, and presence of I*_h_* (Margolis et al., 2006). pDA neurons were voltage clamped at −65 mV, and 300 or 500 nm nicotine was puffed onto target neurons for 10 s (Fig. 2*E2*,*E3*). We noted inward currents in 80% of pDA neurons to both concentrations of nicotine, similar to previous reports (Liu et al., 2012; Engle et al., 2013). This suggests that the concentrations of nicotine used in our CPP assays not only produced reward-related behavior but also were sufficient to activate nAChRs on VTA pDA neurons.

### Upregulation of α4α6*, α4*, and α6* nAChRs in VTA DA neurons

To determine whether nAChR upregulation correlates to nicotine reward-related behavior, we used confocal microscopy assays similar to previous investigations into nAChR upregulation (Henderson et al., 2014, 2016, 2017). Coronal mouse brain slices containing the VTA, SNc, and SNr (bregma, −3.1) were prepared from mice subjected to the CPP assay (Fig. 2*A*,*C*).

As discussed above, we used the presence of α6-GFP to distinguish between DA and GABA neurons in the VTA for our assays using inherent fluorescence (no immunostaining; Fig. 2*A*,*B*). Using FRET methods that have been described previously (Henderson et al., 2017; Avelar et al., 2019), we identified regions of neurons that contain α4α6β2* nAChRs. Upregulation of nAChRs was assessed by quantifying the change in RID of α6-GFP or α4-mCherry fluorescence. We observed a significant increase in the RID of α4α6* nAChRs in VTA DA neurons of both male and female mice used to CPP assays (*p = *0.019 and *p = *0.005 for males and females, respectively; Fig. 2*D1*). We also noted a significant increase in α4* nAChR RID in VTA pDA neurons (*p = *0.05 and *p = *0.008 for males and females, respectively; Fig. 2*D2*). This finding is similar to our previous study, which showed a non-significant increase in α4* nAChR RID using alternating daily injections of 0.5 mg/kg nicotine and saline (Henderson et al., 2017). We observed no change in α6-GFP RID in VTA pDA neurons following nicotine treatment (Fig. 2*D3*). Similar to our CPP assays, we detected no differences between males and females (Table 1).

### Upregulation of α4α6* and α4* nAChRs in VTA pDA neurons correlates to nicotine reward-related behavior

**Figure 3.**
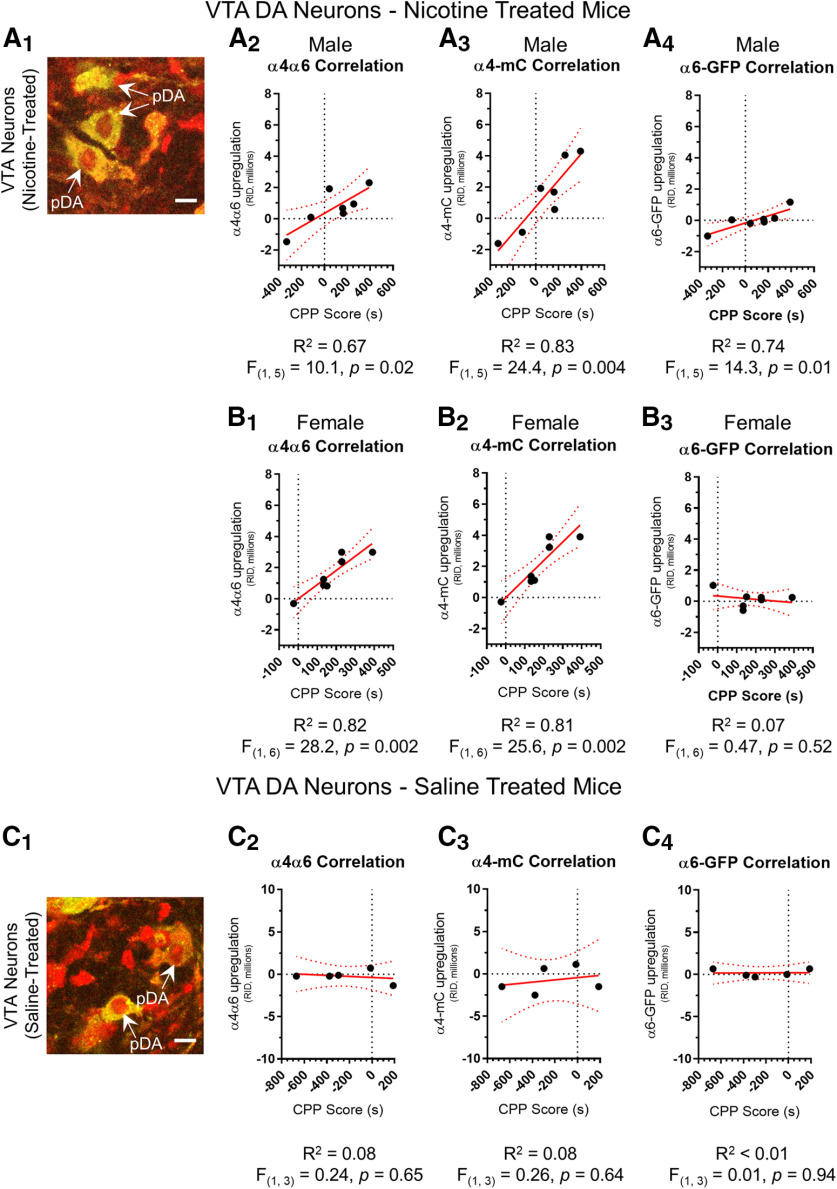
Upregulation of α4α6* and α4* nAChRs in VTA pDA neurons correlates with nicotine reward-related behavior. ***A_1_***, ***C_1_***, Representative merged images of _L_VTA DA neurons in a α4-mCherryα6-GFP brain slice. Scale bar, 10 μm. In nicotine-treated mice, changes in nAChR RID was correlated to CPP score for α4α6* nAChRs, α4* nAChRs, and α6* nAChRs for male (***A_2_***, ***A_3_***, ***A_4_***) and female (***B_1_***, ***B_2_***, ***B_3_***) mice. Linear fits (red line) with 95% confidence intervals (dotted red lines). In saline-treated mice, changes in nAChR RID was correlated to CPP score for α4α6* nAChRs (***C_2_***), α4* nAChRs (***C_3_***) and α6* nAChRs (***C_4_***). Linear fits (red line) with 95% confidence intervals (dotted red lines) were applied using Graphpad Prism software. Nicotine correlations used 7 male and 8 female α4-mCherryα6-GFP mice and the saline correlations used 5 α4-mCherryα6-GFP mice (3 males and 2 females). For each mouse, 36–71 neurons were imaged.*Figure Contributions*: Austin T. Akers, Zachary J. Baumgard, and Brandon J. Henderson performed the experiments and analyzed the data.

Following the observations described above, we analyzed nAChR upregulation for individual mice as a function of their individual CPP score. For each mouse we calculated upregulation scores for α4*, α6*, and α4α6* nAChRs (defined in Materials and Methods), which we then plotted against the individual mouse’s CPP score (Fig. 3*A1–A4,B1–B3*). We found that α4α6* nAChR upregulation in VTA pDA neurons significantly correlated to changes in nicotine reward-related behavior (CPP score) with a *R*^2^ of 0.67 for males (*F*_(1,5)_ = 10.1, *p = *0.02; Fig. 3*A2*) and a *R*^2^ of 0.82 for females (*F*_(1,6)_ = 28.2, *p = *0.002; Fig. 3*B1*). We also observed a significant correlation between α4* nAChR upregulation in VTA pDA neurons and nicotine reward-related behavior. Here, we noted a *R*^2^ of 0.83 (*F*_(1,5)_ = 24.4, *p = *0.004; Fig. 3*A3*) in males and a *R*^2^ of 0.81 (*F*_(1,6)_ = 25.6, *p = *0.002; Fig. 3*B2*) in females. While we observed a significant correlation between α6* nAChR upregulation and nicotine reward-related behavior in male mice (*R*^2^ of 0.74, *F*_(1,5)_ = 14.3, *p *=* *0.01), we note that four of the 6 data points are at the level of zero upregulation. We observed no correlation between α6* nAChR upregulation and reward-related behavior in female mice (Fig. 3*B3*). Using the same methodology, we examined upregulation of the nAChR subtypes in VTA DA neurons from the saline-treated group (Fig. 3*C1–C4*). Here, we found no correlation between changes in nAChR RID and reward-related behavior. These correlative data may imply that the upregulation of α4* and α4α6* nAChRs may underlie nicotine reward-related behavior, at least in the context of CPP assays.

### Upregulation of α4β2* nAChRs on GABA neurons of the VTA and SNr

**Figure 4.**
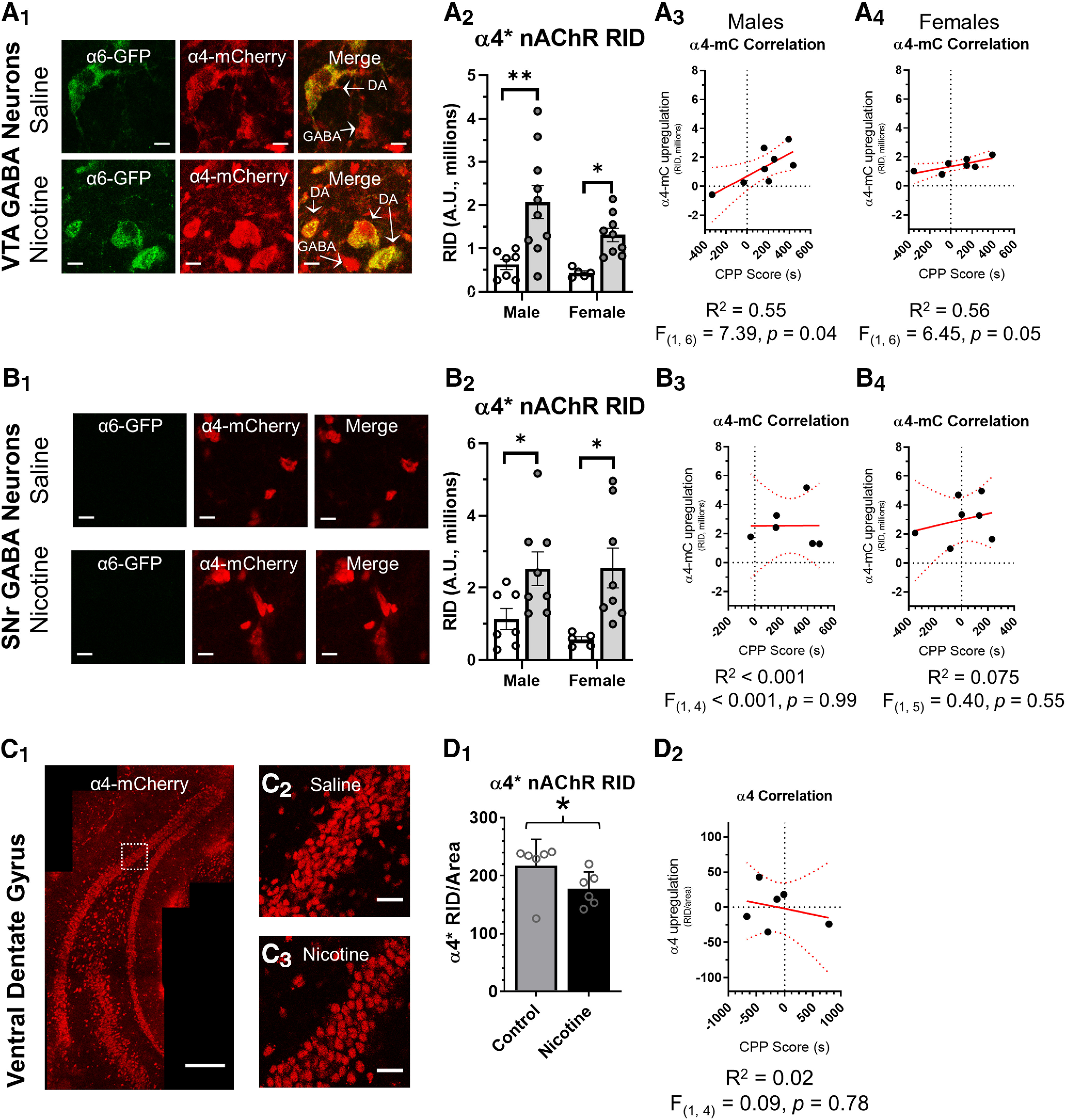
Upregulation of α4* nAChRs on SNr and VTA putative GABAergic neurons may not correlate with nicotine reward-related behavior. ***A_1_***, ***B_1_***, and ***C_1_***, Representative images of neurons in the _L_VTA (***A_1_***), SNr (***B_1_***), or dentate gyrus (***C***) in a α4-mCherryα6-GFP brain slice. Scale bar, 10 μm (***A_1_*** and ***B_1_***), 250 μm (***C_1_***), and 25 μm (***C_2_***, ***C_3_***). ***A_2_***, ***B_2_***, RID of saline- and nicotine-treated mice from CPP assays for SNr GABA neurons or VTA GABA neurons. In ***A_2_*** and ***B_2_*** the n of individual male and female mice are indicated by individual dots. In nicotine-treated mice, changes in nAChR RID was correlated to CPP score for α4* nAChRs in SNr GABA neurons or VTA GABA neurons. In ***A_3_***, ***A_4_***, ***B_3_***, and ***B_4_*** the n of individual male and female mice are indicated by individual dots. ***D_1_***, RID/area of saline- and nicotine-treated mice from CPP assays for dentate gyrus (*n* = 6, 3 male and 3 female). ***D_2_***, Changes in nAChR RID/area was correlated to CPP score for α4* nAChRs in the dentate gyrus. Linear fits (red line) with 95% confidence intervals (dotted red lines) were applied using Graphpad Prism software. Data is Mean ± SEM; *p = 0.05, **p = 0.01; unpaired, two-tailed *t* test. *Figure Contribution:* Austin T. Akers, Zachary J. Baumgard, Skylar Y. Cooper, Alicia J. Avelar, and Brandon J. Henderson performed the experiments and analyzed the data.

The upregulation of α4β2* nAChRs on GABA neurons may play an important role in nicotine’s rewarding effect in the mesolimbic circuit (Mansvelder et al., 2002; Nashmi et al., 2007). Nashmi et al. (2007) showed that α4* nAChRs exhibit a greater degree of upregulation on GABA neurons (VTA and SNr) than they do on DA neurons. In VTA GABA neurons, we observed a significant, 3.4-fold increase in α4-mCherry RID (*p = *0.01; Fig. 4*A1*,*A2*). In male and female mice, we observed a significant correlation between α4* nAChR upregulation in VTA GABA neurons and nicotine reward-related behavior (males, *R*^2^ = 0.55, *F*_(1,6)_ = 7.39, and *p *=* *0.04; and females, *R*^2^ = 0.56, *F*_(1,6)_ = 6.5, *p *=* *0.05; Fig. 4*A3*,*A4*). Similar to the case of α6* nAChR upregulation in male mice, we note that the slope of this correlation is relatively flat.

SNr GABA neurons in mice from the nicotine CPP group exhibited a 2.4-fold increase in α4-mCherry RID that was significant compared with saline-treated mice (*p = *0.02; Fig. 4*B1*,*B2*). While this increase in α4* nAChRs is robust, we observed no correlation between α4* nAChR upregulation in SNr GABA neurons and nicotine reward-related behavior in either male or female mice (Fig. 4*B3*). These data show that concentrations of nicotine sufficient to evoke reward-related behavior upregulate α4β2 nAChRs on GABA neurons in the VTA and SNr. Given the absence of a strong correlation between upregulation and nicotine reward-related behavior, these data also suggest that VTA DA neurons may be more critical for the initiation of nicotine reward. However, this finding needs to be followed-up with functional experiments to examine changes in neurophysiology (see below).

We also investigated how nicotine reward-related behavior is linked to nAChR upregulation in the dentate gyrus (ventral hippocampus; bregma, −3.1; Fig. 4*C1*,*C2*). We observed no significant upregulation in the dentate gyrus of the mice that were part of the nicotine CPP cohort. In fact, we observed a significant decrease in α4* nAChR density (*p *=* *0.047; Fig. 4*D1*). Previous investigations did detect upregulation in the dentate gyrus (Nashmi et al., 2007); but this was with a persistent, high dose of nicotine (2 mg/kg/h via osmotic minipumps for 10 d). Thus, while upregulation is noted with α4* nAChRs in the dentate gyrus with high doses of nicotine, we did not notice upregulation with doses used (0.5 mg/kg injections) to produce reward-related behavior.

### Changes in VTA pDA and putative GABA (pGABA) neuron firing frequency correlates to reward-related behavior

**Figure 5.**
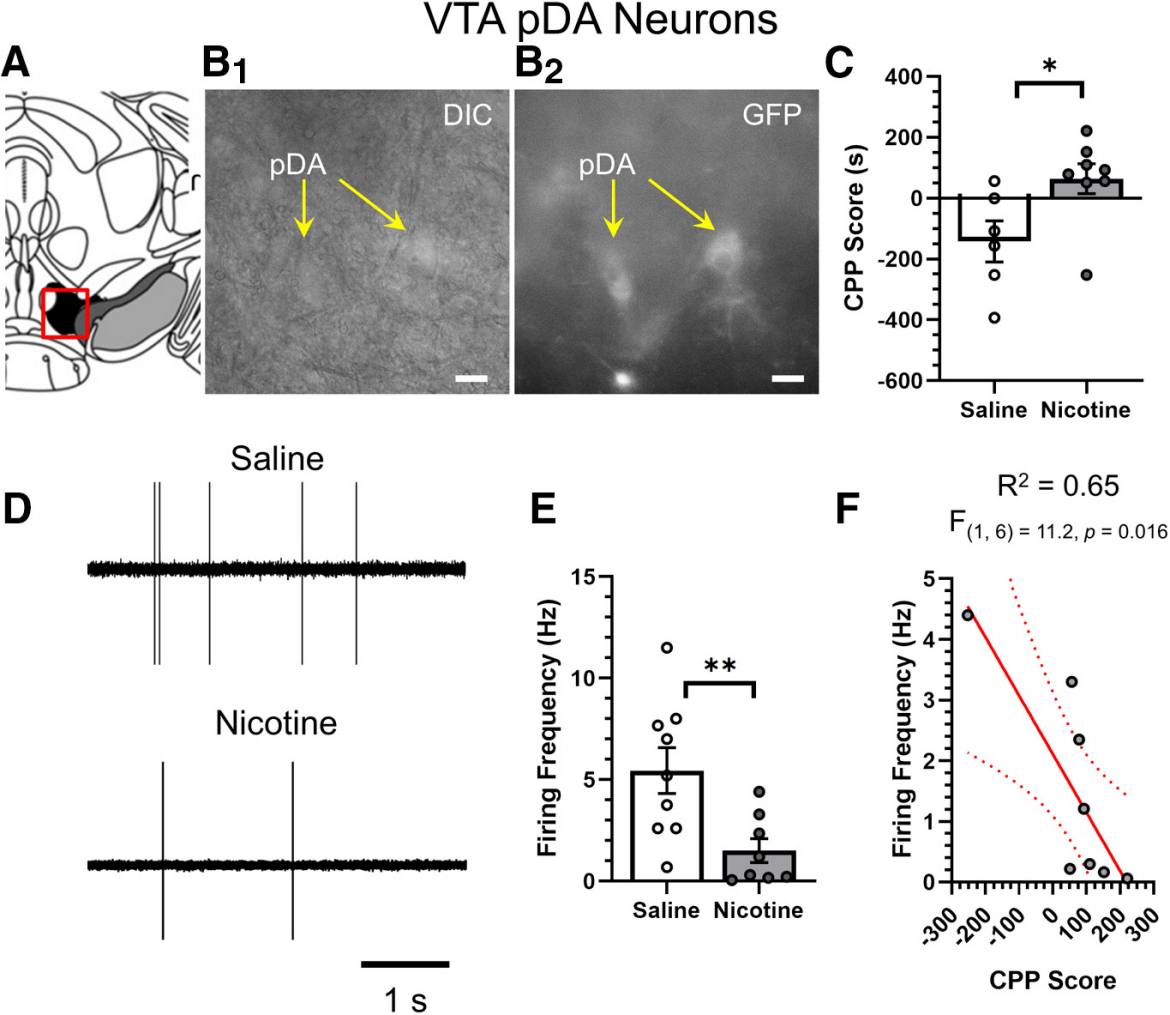
Decreases in VTA pDA neuron firing frequency correlates with reward-related behavior. ***A***, Schematic of target neurons within the _L_VTA (target bregma, −3.1). ***B_1_***, ***B_2_***, Representative images of VTA pDA neurons in DIC (***B_1_***) and GFP (***B_2_***) imaging modes. Scale bar: 20 μm. Mice used in CPP assays (***C***) were used to measure firing frequency of pDA neurons in the VTA (***D***, ***E***). ***D***, Representative cell-attached recordings of VTA pDA neuron baseline firing frequency. ***E***, Mean VTA pDA neuron firing frequency in mice treated with saline or nicotine in CPP assays. Data are mean ± SEM, dots represent data from individual mice (*n* = 8–9 per condition). ***F***, Reward-related behavior (CPP Score) was correlated to baseline firing frequency of _L_VTA pDA neurons; *p <* *0.05, **p <* *0.01; unpaired *t* test. Exact *p* values are given in text. *Figure Contributions*: Brandon J. Henderson performed the experiments and analyzed the data.

While upregulation may indicate changes in nAChR number on neurons, we acknowledge that our confocal methods do not directly indicate whether the upregulation is indicative of increases in intracellular or plasma membrane bound nAChRs. Thus, we used brain-slice electrophysiology to determine whether changes in VTA pDA and pGABA neuron function are linked to reward-related behavior. Nicotine has been observed to decrease the baseline firing frequency of VTA DA neurons and increase the firing frequency of VTA and SNr GABA neurons (Mansvelder et al., 2002; Nashmi et al., 2007). We used a separate cohort of mice assigned to saline or nicotine in CPP assays (Fig. 5*C*). Similar to results described above, we noted a significant difference between saline-treated and nicotine-treated mice in our CPP assay (*p *=* *0.02). Similar to microscopy assays, at the end of CPP, mouse brains were extracted but here were used for brain-slice electrophysiology assays. Similar to microscopy assays, we used α6-GFP fluorescence to identify pDA neurons in the _L_VTA (Fig. 5*A*,*B1*,*B2*). Using cell-attached configurations we examined baseline firing frequency of VTA pDA neurons (Fig. 5*D*). Here, we observed that nicotine-treated mice exhibited a significant decrease in baseline firing of VTA pDA neurons (*p *=* *0.009; Fig. 5*E*) that matches previous investigations (Nashmi et al., 2007; Xiao et al., 2009). Next, we correlated the VTA pDA neuron firing frequency of individual nicotine-treated mice (mean of ≥3 neurons per mouse) to its respective CPP score (Fig. 5*F*). We observed a significant inverse-correlation between firing frequency of VTA pDA neurons and nicotine reward-related behavior (*R*^2^ = 0.65, *F*_(1,6)_ = 11.2, *p *=* *0.016).

**Figure 6.**
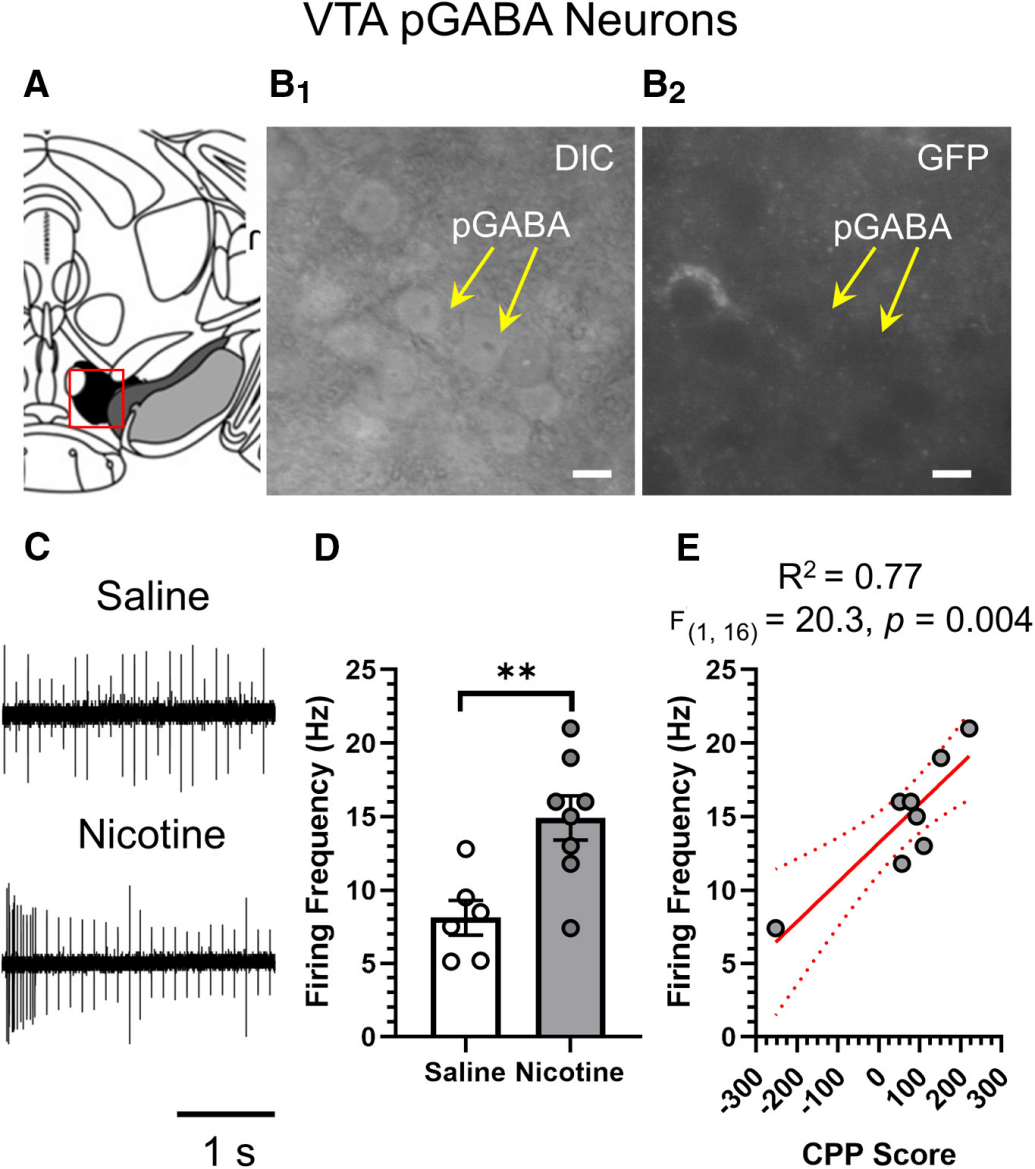
Increase in VTA pGABA neuron firing frequency correlates with reward-related behavior. ***A***, Schematic of target pGABA neurons within the _L_VTA (target bregma, −3.1). ***B_1_***, ***B_2_***, Representative images of VTA pGABA neurons in DIC (***B_1_***) and GFP (***B_2_***) imaging modes. Scale bar: 20 μm. ***C***, Representative cell-attached recordings of VTA pGABA neuron baseline firing frequency from mice assigned to saline or 0.5 mg/kg nicotine CPP cohorts. ***D***, Mean VTA pGABA neuron firing frequency in mice treated with saline or nicotine in CPP assays. Data are mean ± SEM ***E***, Reward-related behavior (CPP Score) were correlated to baseline firing frequency of VTA pGABA neurons; **p < 0.01 with; unpaired *t* test. Exact *p* values are given in text. *Figure Contributions*: Brandon J. Henderson performed the experiments and analyzed the data.

Using these same mice, we examined the firing frequency of VTA pGABA neurons (Fig. 6*A*,*B1*,*B2*). Similar to previous reports, we noted a significant increase in the baseline firing frequency of VTA pGABA neurons in mice assigned to the nicotine CPP cohort (Fig. 6*D*). Despite observing no significant correlation between nicotine reward-related behavior and α4β2 nAChR upregulation in VTA GABA neurons (or SNr GABA neurons), we did observe a significant correlation between nicotine reward-related behavior and VTA pGABA neuron firing frequency (*R*^2^ = 0.77, *F*_(1,16)_ = 20.3, *p *=* *0.004; Fig. 6*E*).

## Discussion

It is important to note that nAChRs on VTA DA neurons would be functionally activated as our data (300 and 500 nm nicotine; Fig. 2) and previous investigations (300 nm; Liu et al., 2012; Engle et al., 2013) show that nicotine concentrations that match those present in CPP assays are sufficient to evoke inward currents in VTA DA neurons. These previous reports also stress that activation with this low concentration of nicotine only activates nAChRs that contain α4 and α6 subunits. In line with these previous studies, we noted a significant link between nicotine reward-related behavior and α4α6* nAChRs, α4(non-α6)* nAChRs, but not α6(non-α4)* nAChRs, in the _L_VTA. We observed no relationship between α4* nAChR upregulation in the SNr to nicotine reward-related behavior. This finding matches a previous investigation by Pons et al. (2008) that showed nicotine self-administration in mice depends on α4*, α6*, and β2* nAChRs in the VTA but not in the substantia nigra.

One limitation of the current study is that we only observed a single time point (the final CPP test day). The problem this poses is that our analysis of upregulation and neuronal firing frequency captures the target nAChRs in a state that is free of nicotine for ∼24 h. As a result, even in this case where we observed nAChR upregulation and changes in firing frequency in our assays, we ca not adequately assess how nicotine-induced activation of the nAChRs connects to our observed reward-related behavior. Furthermore, our behavioral assay (CPP) is a non-contingent assay and while it provides information regarding nicotine-related reward, it does not provide insight into reinforcement or the impact of long-term nicotine exposure. One manner to improve on this would be to use a contingent drug delivery assay (intravenous or vapor self-administration). With these assays we would be able to extract the brains immediately after operant responding so that we can capture the behavioral endpoint and the brain samples that are still saturated with nicotine. Thus, we could examine changes to nAChRs while nicotine is present and use a model that more closely matches smoking-related behaviors. These assays (vapor self-administration) are currently being pursued but are only at their early stages.

Previous reports show that nicotine robustly upregulates high-sensitivity α4β2 nAChRs on SNr and VTA GABAergic neurons (Nashmi et al., 2007; Xiao et al., 2009), and we have reproduced that here (Fig. 4). Interestingly, our data show a link between α4* nAChR upregulation on VTA pGABA neurons and nicotine reward-related behavior but no correlation between upregulation of α4* nAChRs on SNr pGABA neurons and reward-related behavior. For α4β2 nAChR upregulation on VTA GABA neurons, the *R*^2^ values of 0.55 and 0.56 (males and females, respectively) may indicate their upregulation is less sensitive when compared with what we observed on pDA neurons. Here, we must also take into consideration that pDA neurons have a multitude of nAChR subtypes that assemble in multiple possible stoichiometries [α4β2 (α4_(3)_β2_(2)_ and α4_(2)_β2_(3)_), α4α6β2 (α4_(1)_α6_(1)_β2_(3)_, α4_(2)_α6_(1)_β2_(2)_, α4_(1)_α6_(2)_β2_(2)_, α4_(1)_α6_(1)_β2_(2)_β3_(1)_, and potentially others), and α6β2* (mostly α6_(2)_β2_(2)_β3_(1)_)]. Thus, we must acknowledge that changes in RID of nAChR subunits may also be the result of a net change in stoichiometry. This is another weakness of our methods as we cannot distinguish between nAChRs that reside on the plasma membrane from those that are intracellular. Additionally, we can detect changes in stoichiometry but not define specific stoichiometries. The former problem (plasma membrane changes) is partially reconciled by our electrophysiology assays (discussed below) but the latter can now be addressed using newly developed single-molecule microscopy assays (Fu et al., 2019).

Our electrophysiological methods detected a correlation between VTA pGABA neurons and reward-related behavior that is greater than the correlation between VTA pDA neuron firing and reward-related behavior. One conclusion for this is that our electrophysiological methods are more sensitive than that of our microscopy assays. While this is likely true, the caveat is that brain-slice electrophysiology is lower throughput as we can examine 4–10 neurons per mouse, while there are hundreds of neurons in each brain region that could be measured using microscopy. Consequently, the electrophysiological methods may be a more accurate determination of changes in neuronal populations in relation to reward-related behavior.

Following recent investigations into VTA DA and GABA neurons (Grieder et al., 2019), a second conclusion is that there is a distinct difference in the role that DA and GABA systems play within the VTA in nicotine reward. The work by Grieder et al. (2019) revealed that VTA DA neurons mediate aversion while VTA GABA neurons mediate reward to nicotine. This finding also agrees with another previous report that revealed the selective upregulation of α4β2* nAChRs on VTA GABA neurons was sufficient to produce CPP in mice and increased sensitivity to nicotine reward (Ngolab et al., 2015). Our observations of an inverse-correlation between VTA pDA neuron firing and nicotine reward but a positive correlation between VTA pGABA neuron firing and nicotine reward may support these previous conclusions. One way to address distinction between aversive and rewarding stimuli within the VTA with our current methodology would be to repeat these microscopy and electrophysiology assays but with a conditioned place aversion paradigm using a higher, aversive dose of nicotine.

While this study focuses on DA and GABA neurons of the midbrain, there are other cell types and brain regions that are critical to nicotine reward and reinforcement. Accordingly, our understanding of how changes in nAChR upregulation and function directly link to reward (or reinforcement) is still in the beginning stages and have yet to examine the recently defined local nicotine-related glutamatergic inputs (Yan et al., 2018, 2019) or the aversive stimuli that originate from the habenula and interpeduncular nuclei (Fowler et al., 2011; Fowler and Kenny, 2012). The methods of this study should be applied to several additional behavioral aspects of chronic nicotine, namely nicotine self-administration. At present, our data show that the upregulation of α4* and α4α6* nAChRs in VTA neurons is tied to nicotine reward-related behavior.

## Synthesis

Reviewing Editor: Christie Fowler, University of California, Irvine Decisions are customarily a result of the Reviewing Editor and the peer reviewers coming together and discussing their recommendations until a consensus is reached. When revisions are invited, a fact-based synthesis statement explaining their decision and outlining what is needed to prepare a revision will be listed below. The following reviewer(s) agreed to reveal their identity: Jessica Ables.

Both reviewers felt that the results are interesting and important for the field. However, several significant concerns were noted. First, it needs to be addressed as to why GABAergic VTA and SNr neurons did not correlate with CPP given that alpha4 upregulation is robust in VTA and SNr GABAergic neurons. Second, in addition to the pooled data, the upregulation and correlations should also be separated according to sex to determine if males and females differ in these outcome measures. This may shed light on the weak correlation between alpha4 upregulation and CPP in GABA neurons. Third, it was found that alpha4 did increase with nicotine in VTA dopaminergic neurons, but it was not significant. Adding more subjects in this group to determine whether there is a significant correlation, rather than a very weak trend (p=0.12), would strengthen the overall findings. Finally, concerns were noted regarding the statistical approach, statistical reporting, and information provided in the methods (see below individual reviews).

Reviewer 1:

In the manuscript “Upregulation of α4* and α4α6* nAChRs on VTA dopamine neurons is correlated with nicotine reward-related behavior", the authors used transgenic mice (over)expressing fluorescent-tagged nAChRs to examine upregulation of specific nAChRs on DA and GABAergic neurons in the VTA, as well as to determine what role those receptors play in the early stages of nicotine reward. Overall I found the manuscript to be logically organized and the experiments well designed to answer the proposed hypothesis. The findings that 3 doses of nicotine were sufficient to upregulate nAChRs and alter function of VTA DA and GABAergic neurons is interesting to the addiction field as a whole, and highlights the need to study the early stages of drug dependence. However, there are some minor points that should be addressed to significantly improve the manuscript, particularly for clarity of methods.

1. The manuscript, in particular the methods, should be improved for clarity.

a. It is not clear whether the reported doses for nicotine are tartrate or free base. Menthol is included in the reagents, but no menthol is used in the experiments as presented. In e-phys methods, nicotine application in slice is stated as 300-500 uM, while results/discussion states 300-500 nM. Please clarify.

b. It would be helpful to include a bit more detail or RRID for the mice used in this study, similar to referenced paper (Henderson 2016): “The α6-eGFP bacterial artificial chromosome (BAC) transgenic strain used in this study possessed six copies of the α6-eGFP BAC and has been described previously (Mackey et al., 2012; Henderson et al., 2014). The α4-mCherry knock-in mouse strain and the β3-GFP nAChR knock-in mouse strain have been described previously (Shih et al., 2014; Srinivasan et al., 2016).” What is the rationale for using transgenic (presumably hemizygyous, BAC, 6 copies) a6-GFP and homozygous a4-mCherry (knock-in)?

c. The methods state: “An unbiased protocol was used where nicotine was given in the white/grey chamber on ‘drug’ days and saline was given in the white/black chamber on saline days.” This is confusing in context with the next sentence that describes multiple pairing session per day. Simply state that nicotine was paired with the [white] chamber and saline with the [black] chamber. State which chamber is neutral and whether animals were allowed access to the neutral chamber during pairing sessions. Likewise, “Data are expressed as a change in baseline preference between the pre- and post-test.” Remove “baseline” to indicate that the preference score is calculated as the difference between pre- and post-test.

d. Please provide dilutions for antibodies, primary and secondary, used for IHC.

2. In figure 1A, the background appears quite high, such that the IPN appears to be costained for GFP and TH, which is not to be expected. TH staining should not appear in the IPN.

3. Can you provide an orthogonal projection for 1B to demonstrate colocalization of signals?

4. Experimentally, I have the most difficulty reconciling the finding that a4-mC on VTA GABA neurons does not correlate with CPP score (Fig 4A3), despite the significant increase in expression and the electrophysiological correlation. There is a trend toward significance, p=0.12, and I wonder if adding more animals might improve this finding. Likewise, could the GABAergic neurons be broken down by subnuclei and might that improve the correlation?

Reviewer 2:

In this study, the authors examined whether there was a correlation between nAChR upregulation (a4 subunits, a6 subunits or a4a6* receptors) in VTA GABAergic or dopaminergic neurons with nicotine mediated reward behaviour as measured with CPP and whether the nicotine mediated reward behaviour of CPP correlated with the firing of dopaminergic or GABAergic neurons.

Some aspects have already been reported previously such as nicotine induced upregulation of a4, nicotine’s effect on a4a6* receptors and nicotine’s effect on neuronal excitability. Hence, the results of this study confirm previous studies by the authors and others. However, the novel aspect of this study was combining with reward like behaviour and correlating site specific upregulation with reward and also site specific changes in neuronal excitability with reward to determine through correlation which upregulated subunits in which neurons and which change in neuronal activity may be contributing to nicotine mediated reward like behaviour.

Overall this is an interesting study that was carefully performed and the results are believable.

Major Issues

1) Since experiments were performed on both male and female mice, it would be informative to also show the results for male only and female only in addition to the pooling of both sexes as was done in the study.

2) The result of the a4a6* receptor upregulation is interesting and was nicely determined using FRET technique. However, the authors should be cautioned in interpreting the increased FRET signal solely as upregulation of a4a6* receptors. There should be something in the discussion including alternative interpretations. For example, the increased FRET may involve a change in stoichiometry of a4a6* receptors. This may be a possibility since a6 subunits did not upregulate but a4 did with repeated injections of nicotine.

3) For the statistics of upregulation of nAChRs unpaired t tests were used. Did the authors check that the data were normally distributed in order to meet the assumptions of using the t test? If the data are not normally distributed, nonparametric test such as the Wilcoxon rank sum test otherwise known as the Mann Whitney test can be used on non-normal data.

4) In the Methods, the authors need to mention the software used to analyze and determine the raw integrated intensity. Also details were lacking whether all the neurons from each brain section of the VTA and SNr analyzed or were a subpopulation randomly selected?

Minor Issues

1) In the methods page 5, the authors describe that imaging was done with a Leica SP5 TCSII confocal microscope and the subheading was entitled “Spectral confocal imaging of mouse brain slices”. I want to confirm whether spectral confocal microscopy imaging was indeed performed or if otherwise then the subheading should be changed to “Confocal imaging of mouse brain slices”.

## Author Response

Reviewer 1:

In the manuscript “Upregulation of α4* and α4α6* nAChRs on VTA dopamine neurons is correlated with nicotine reward-related behavior", the authors used transgenic mice (over)expressing fluorescent-tagged nAChRs to examine upregulation of specific nAChRs on DA and GABAergic neurons in the VTA, as well as to determine what role those receptors play in the early stages of nicotine reward. Overall I found the manuscript to be logically organized and the experiments well designed to answer the proposed hypothesis. The findings that 3 doses of nicotine were sufficient to upregulate nAChRs and alter function of VTA DA and GABAergic neurons is interesting to the addiction field as a whole, and highlights the need to study the early stages of drug dependence. However, there are some minor points that should be addressed to significantly improve the manuscript, particularly for clarity of methods.

1. The manuscript, in particular the methods, should be improved for clarity.

a. It is not clear whether the reported doses for nicotine are tartrate or free base. Menthol is included in the reagents, but no menthol is used in the experiments as presented. In e-phys methods, nicotine application in slice is stated as 300-500 uM, while results/discussion states 300-500 nM. Please clarify.

Response: This has been clarified in the revised Methods and Results sections.

b. It would be helpful to include a bit more detail or RRID for the mice used in this study, similar to referenced paper (Henderson 2016): “The α6-eGFP bacterial artificial chromosome (BAC) transgenic strain used in this study possessed six copies of the α6-eGFP BAC and has been described previously (Mackey et al., 2012; Henderson et al., 2014). The α4-mCherry knock-in mouse strain and the β3-GFP nAChR knock-in mouse strain have been described previously (Shih et al., 2014; Srinivasan et al., 2016).” What is the rationale for using transgenic (presumably hemizygyous, BAC, 6 copies) a6-GFP and homozygous a4-mCherry (knock-in)?

Response: The reason why we use a transgenic α6-GFP but a knock-in (HOM) for α4-mCherry is simply because these two mouse lines are already available and have been used previously to specifically identify and examine nAChRs that contain both the α4 and α6 nAChR subunit. Currently, there are no knock-in α6-GFP mice or transgenic α4-mCherry mice available. Thus, using another genetically modified mouse line to examine both α6* and α4* nAChRs would require a significant amount of time to derive a new mouse colony.

c. The methods state: “An unbiased protocol was used where nicotine was given in the white/grey chamber on ‘drug’ days and saline was given in the white/black chamber on saline days.” This is confusing in context with the next sentence that describes multiple pairing session per day. Simply state that nicotine was paired with the [white] chamber and saline with the [black] chamber. State which chamber is neutral and whether animals were allowed access to the neutral chamber during pairing sessions. Likewise, “Data are expressed as a change in baseline preference between the pre- and post-test.” Remove “baseline” to indicate that the preference score is calculated as the difference between pre- and post-test.

Response: We have made the suggested changes in the revised manuscript.

d. Please provide dilutions for antibodies, primary and secondary, used for IHC.

Response: We used 1:500 and 1:1000 concentrations of primary and secondary antibodies, respectively. We have added this to our revised methods section.

2. In figure 1A, the background appears quite high, such that the IPN appears to be costained for GFP and TH, which is not to be expected. TH staining should not appear in the IPN.

Response: Thank for your attention on this issue. The α4-mCherryα6-GFP mice do possess a high level of background in the red emission range due to the widespread expression of α4-mCherry. To fix this we utilized secondaries in the blue-range as opposed to red. The revised staining has little to no immunofluorescence in the IPN and SNr (see revised Figure 1).

3. Can you provide an orthogonal projection for 1B to demonstrate colocalization of signals?

Response: We have not provided orthogonal projections of our signals but have added a new table that indicates the number of neurons in the VTA and SNc that overlap between TH and GFP immunofluorescence (Table 2).

4. Experimentally, I have the most difficulty reconciling the finding that a4-mC on VTA GABA neurons does not correlate with CPP score (Fig 4A3), despite the significant increase in expression and the electrophysiological correlation. There is a trend toward significance, p=0.12, and I wonder if adding more animals might improve this finding. Likewise, could the GABAergic neurons be broken down by subnuclei and might that improve the correlation?

Response: In conducting additional experiments to separate males and females, VTA GABA neurons in both males and females exhibit a correlation between α4* nAChR upregulation and nicotine reward-related behavior (see revised Figure 4A1-4). Despite the upregulation of nAChRs in the SNr we did not detect a relationship between upregulation in the SNr and reward-related behavior. Accordingly, this has led to significant changes in our discussion section.

Reviewer 2:

In this study, the authors examined whether there was a correlation between nAChR upregulation (a4 subunits, a6 subunits or a4a6* receptors) in VTA GABAergic or dopaminergic neurons with nicotine mediated reward behaviour as measured with CPP and whether the nicotine mediated reward behaviour of CPP correlated with the firing of dopaminergic or GABAergic neurons.

Some aspects have already been reported previously such as nicotine induced upregulation of a4, nicotine’s effect on a4a6* receptors and nicotine’s effect on neuronal excitability. Hence, the results of this study confirm previous studies by the authors and others. However, the novel aspect of this study was combining with reward like behaviour and correlating site specific upregulation with reward and also site specific changes in neuronal excitability with reward to determine through correlation which upregulated subunits in which neurons and which change in neuronal activity may be contributing to nicotine mediated reward like behaviour.

Overall this is an interesting study that was carefully performed and the results are believable.

Major Issues

1) Since experiments were performed on both male and female mice, it would be informative to also show the results for male only and female only in addition to the pooling of both sexes as was done in the study.

Response: For all CPP and microscopy assays we have now separated the data to show the males and females separately.

2) The result of the a4a6* receptor upregulation is interesting and was nicely determined using FRET technique. However, the authors should be cautioned in interpreting the increased FRET signal solely as upregulation of a4a6* receptors. There should be something in the discussion including alternative interpretations. For example, the increased FRET may involve a change in stoichiometry of a4a6* receptors. This may be a possibility since a6 subunits did not upregulate but a4 did with repeated injections of nicotine.

Response: This is an excellent point and we have now included this into our discussion.

3) For the statistics of upregulation of nAChRs unpaired t tests were used. Did the authors check that the data were normally distributed in order to meet the assumptions of using the t test? If the data are not normally distributed, nonparametric test such as the Wilcoxon rank sum test otherwise known as the Mann Whitney test can be used on non-normal data.

Response: In revising our manuscript and examining both males and females separately we have changed to a two-way ANOVA to determine the statistical significance of our nAChR upregulation data.

4) In the Methods, the authors need to mention the software used to analyze and determine the raw integrated intensity. Also details were lacking whether all the neurons from each brain section of the VTA and SNr analyzed or were a subpopulation randomly selected?

Response: We did not select subpopulations of brain regions to analyze as we believed this would bias our data analysis process. Instead, we imaged and analyzed all the GFP and/or mCherry neurons that were present in every brain region of each brain slice. We have included this addition, as well as the software used, in our revised methods.

Minor Issues

1) In the methods page 5, the authors describe that imaging was done with a Leica SP5 TCSII confocal microscope and the subheading was entitled “Spectral confocal imaging of mouse brain slices”. I want to confirm whether spectral confocal microscopy imaging was indeed performed or if otherwise then the subheading should be changed to “Confocal imaging of mouse brain slices”.

Response: Thank you for your attention, indeed the SP5 TCSII does not include spectral detection capabilities. We have changed the title of this subsection as you have indicated.
